# Case report: Advanced modified pneumatic retinopexy for treating simultaneous bilateral rhegmatogenous retinal detachment

**DOI:** 10.3389/fmed.2024.1370739

**Published:** 2024-06-26

**Authors:** Shenchao Guo, Tiepei Zhu, Xin Zhang, Gaochun Li, Zhenyang Xiang, Enhui Li

**Affiliations:** ^1^Department of Ophthalmology, Taizhou Hospital of Zhejiang Province, Taizhou, China; ^2^Eye Center, Second Affiliated Hospital of Medical College, Zhejiang University, Hangzhou, China

**Keywords:** rhegmatogenous retinal detachment, pneumatic retinopexy, gas injection, head position maneuver, bilateral

## Abstract

**Background:**

Simultaneous bilateral rhegmatogenous retinal detachment (RRD) is a rare and challenging condition in ophthalmology. This case report focuses on a modified pneumatic retinopexy technique, designed to improve treatment outcomes for this difficult condition.

**Case presentation:**

A 59-year-old male presented with decreased visual acuity in his right eye for one week. Examination revealed extensive retinal detachment in the right eye with multiple superior breaks and macula off, separated by approximately 3 clock hours. The left eye exhibited one quartile of retinal detachment with superior breaks and macula on. Bilateral simultaneous PR was performed for retinal repair. In the modified PR procedure, 0.7 ml of low-concentration perfluoropropane and 0.7 ml of filtered pure air were intravitreally injected into the right and left eyes, respectively. A head position maneuver was then employed to sequentially close retinal breaks, followed by laser photocoagulation once the surrounding retina reattached. Two days after gas injection, both retinas were completely reattached. Best corrected visual acuity improved to 0.6 in the right eye and 0.9 in the left eye at the 8-month follow-up.

**Conclusion:**

The innovative modified pneumatic retinopexy technique presented in this case report offers a promising new approach for effectively treating simultaneous bilateral rhegmatogenous retinal detachment.

## Introduction

Bilateral rhegmatogenous retinal detachment (RRD), characterized by the simultaneous detachment of the neural retina from the underlying pigment epithelium in both eyes, is a rare occurrence, representing a fraction of retinal detachment cases. However, when it does occur, it poses a significant threat to vision and quality of life, necessitating prompt and effective treatment. The conventional approach to RRD, which often involves scleral buckling, vitrectomy, or standard pneumatic retinopexy, might not always be suitable or sufficiently effective for bilateral cases due to the complexity and variation in each eye’s detachment (1, 2). PR offers advantages in terms of reduced invasiveness, quicker visual recovery, and a lower complication risk compared to scleral buckling and pars plana vitrectomy (3, 4). It is applied in uncomplicated RRD cases, with eligibility criteria encompassing a single break or multiple breaks separated by one clock hour, preferably located within the superior eight clock hours of the retina (4). However, traditional PR, as an office-based technique, may encounter challenges in achieving successful retinal reattachment in cases of bilateral simultaneous RRD due to diverse locations and types of retinal breaks in both eyes, particularly when multiple retinal breaks are separated by approximately 3 clock hours.

Previously, our studies presented an inpatient PR procedure for RRD patients, demonstrating a notably high success rate in retinal reattachment (5). This report outlines a modified PR technique employing a head position maneuver that effectively managed a case of simultaneous bilateral RRD, even when one eye exceeded the classic indications of traditional PR.

## Case presentation

A 59-year-old male presented to our department with reduced vision in his right eye persisting for a week. On examination, the best corrected visual acuity (BCVA) was 0.02 in the right eye and 0.7 in the left eye. The slit lamp examination indicated an unremarkable anterior segment and mild nuclear sclerosis in both eyes. Dilated fundus examination revealed a substantial superior retinal detachment extending from 8:00 to 4:00 in the right eye, marked by multiple breaks and macular detachment, with a significant tear at 11:00 and several smaller holes at 1:30. Additionally, linear lattice degeneration with a small atrophic hole was observed in the peripheral inferotemporal retina from 7:00 to 8:00. Incidentally, the left eye exhibited a quarter retinal detachment from 11:00 to 2:00, multiple retinal breaks at 1:00, with the macula remaining attached. While the left eye met the typical criteria for PR, the right eye presented a complex retinal detachment with breaks spanning over one clock hour, making it unsuitable for traditional PR. However, a modified PR approach involving head positioning maneuvers was considered viable for the right eye. Subsequent treatment discussions led to the patient opting for immediate bilateral simultaneous PR for both eyes.

Following hospitalization, laser photocoagulation targeted any peripheral lattice degeneration in the attached retina before PR. A two-step PR method involving laser retinopexy for primary retinal detachment repair was employed. During the surgery, the right eye was operated on first, involving paracentesis to cautiously remove at least 0.5 mL of aqueous solution. This was accomplished by using a 1 ml syringe connected to a 30-gauge needle. While the needle was inserted into the anterior chamber, pressure was applied to the eyeball using a cotton swab, allowing for the slow release of fluid from the vitreous cavity or posterior chamber into the anterior chamber. Subsequently, 0.7 mL of a low concentration of perfluoropropane (0.1 mL of C_3_F_8_ and 0.6 mL of filtered pure air) was injected into the vitreous via the pars plana using a 30-gauge needle. Post-gas injection, intraocular pressure was checked to ensure normal perfusion of the central retinal artery. A similar procedure was performed on the left eye, although 0.7 mL of filtered pure air without perfluoropropane was chosen for gas injection.

**FIGURE 1:**
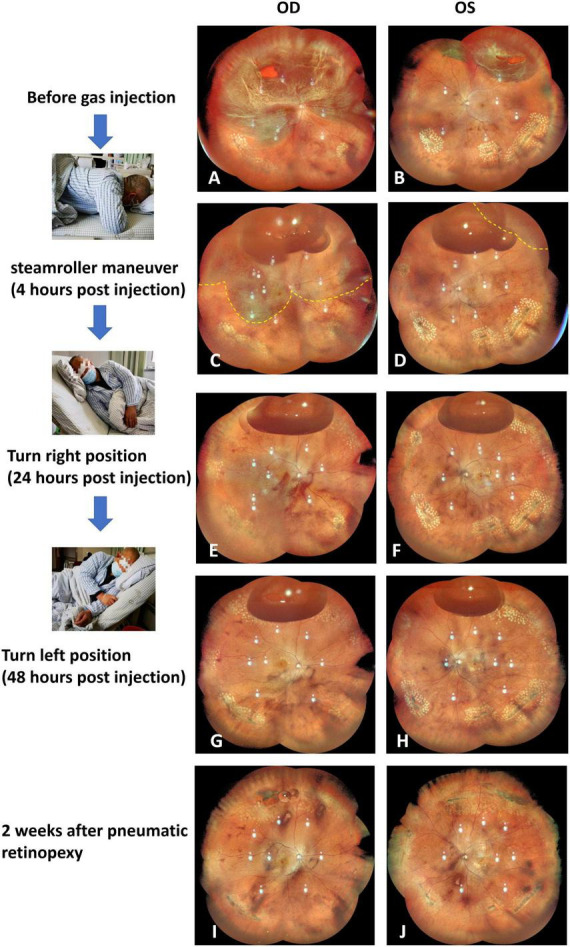
Ultrawide fundus photographs depict the progressive retinal reattachment in the right and left eyes before the PR surgery **(A,B)**, after the steamroller head position maneuver at 4 h post gas injection **(C,D)**, after turning right and maintaining a semi-recumbent posture at 24 h post gas injection **(E,F)**, after maintaining a semi-recumbent posture on the left side at 48 h post gas injection **(G,H)**, and at two weeks after PR treatment **(I,J)**, respectively.

**FIGURE 2:**
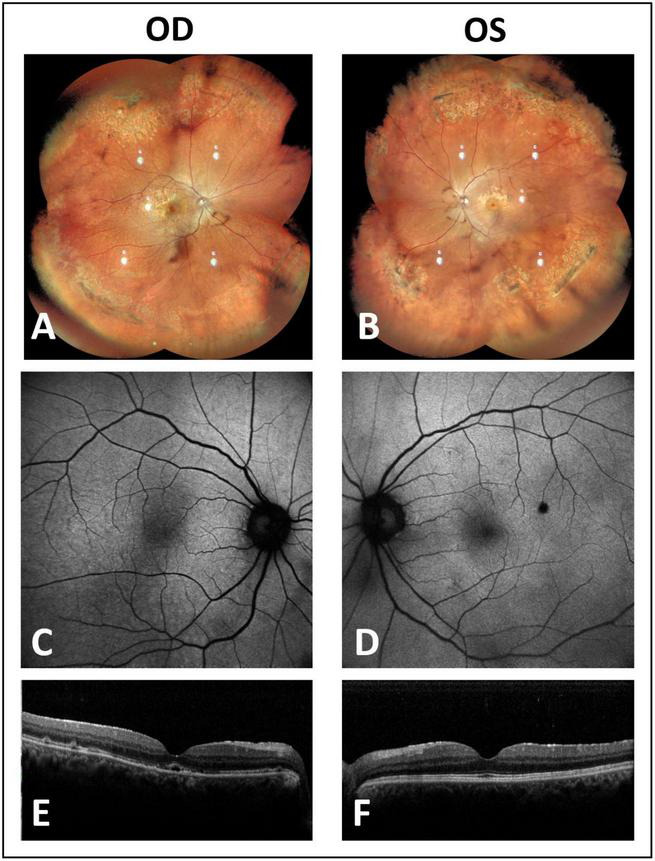
At 8 months post-PR treatment, examination via fundus photography revealed the reattachment of previously detached retinas **(A,B)**. Fundus autofluorescence imaging exhibited an absence of vessel printing **(C,D)**, and optical coherence tomography images showcased the restoration of outer retinal structures **(E,F)**.

The patient received immediate instructions for a specific head position maneuver (Figure 1). Initially, the patient’s head was positioned face-down for an hour to allow the gas bubble to displace subretinal fluid away from the macula in the right eye and prevent macular detachment in the left eye. To prevent potential complications arising from the close proximity of inferotemporal lattice degeneration to the detached retina in the right eye, a left-lateral recumbent position was adopted, facilitating the movement of the gas bubble to the temporal junction of the attached and detached retina. A specialized technique, known as the steamroller technique, was employed to expel temporal subretinal fluid through the open retinal break in the right eye. The head elevation occurred in increments of 30° every hour during the steamroller maneuver until the large retinal break was positioned upward. After three hours, the temporal peripheral retina in the right eye and a majority of the retina in the left eye had achieved attachment, accompanied by a significant reduction in subretinal fluid. Afterward, the patient was advised to turn right and maintain a semi-recumbent posture to enable the gas bubbles to block the retinal breaks at 1:00 in the left eye and the small punctures at 1:30 in the right eye. The next day, the reverberation of the left eye’s superotemporal retina and the right eye’s superonasal retina had taken place entirely. This was followed by laser photocoagulation being employed around the breaks in both reattached retinas. While the right eye’s superotemporal and posterior retina were still incompletely reattached, the patient was instructed to switch position and maintain a semi-recumbent posture on his left side to treat the enormous retinal tear at 11:00. On the second day after the gas injection, the right eye’s retina had become wholly reattached. Furthermore, the significant retinal tear at 11:00 was dealt with using laser photocoagulation. Changing positions on the left and right sides was sustained for five days. The gas bubble in the right eye persisted for nearly two weeks after injection. At the 8-month follow-up, both retinas remained attached, confirmed by fundus autofluorescence illustrating no evidence of retinal displacement (Figure 2). Optical coherence tomography images indicated restored outer retinal structures, with BCVA improving to 0.6 in the right eye and 0.9 in the left eye, without any postoperative complications noted during subsequent follow-ups.

## Discussion

In this study, we presented a case of simultaneous PR with a head positioning maneuver for a patient experiencing bilateral simultaneous RRD. Successful reattachment of the retinas was achieved through a single PR treatment for each eye. The right eye in our case did not conform to typical indications for PR, as it exhibited breaks separated by approximately 3 clock hours and extensive lattice degeneration. Our report also introduces modifications to the PR procedure, including the use of different gas types for intravitreal injection and the application of a head positioning maneuver to address separated breaks and various regions of detached retina. Notably, no intraoperative or postoperative complications were observed in this case.

To date, only two case reports have demonstrated simultaneous PR for bilateral RRD [Kerimoglu et al. (6) and Rubin et al. (7)]. In our case report, we deviated from previous approaches. We employed a dynamic head positioning maneuver instead of a fixed head position to manage multiple retinal breaks and small atrophic holes in the inferior lattice degeneration. Initially, the patient turned to the left side, and the steamroller technique was executed to prevent temporal subretinal fluid from extending to the inferior lattice degeneration, which could induce reopening of atrophic holes leading to new retinal detachment. Subsequently, the patient underwent a series of head position changes over the next 48 h until the retinas were completely reattached. During these maneuvers, the gas bubble was strategically moved to prioritize the treatment of simple retinal detachment with macula-on in the left eye and to block multiple small holes before addressing the large retinal tear in the right eye. The stepwise process of blocking retinal breaks and applying laser photocoagulation as soon as possible upon reattachment was crucial. Another distinctive aspect of our case report was the choice of gas for intravitreal injection. Filtered pure air was injected for the left eye with simple retinal detachment, while a low concentration of C_3_F_8_ (14%) was used for the right eye with complicated retinal detachment. Notably, this report is the first to introduce the use of a low concentration of expansile gas for PR. The 14% C_3_F_8_ provided a moderate duration of intravitreal gas bubbles, maintaining almost unchanged volume during the first three days after injection, ensuring sufficient buoyancy and surface tension to block retinal breaks during the head position maneuver.

The traditional PR is usually suitable for simple retinal detachment cases with superior retinal break or multiple breaks within a 1-clock-hour range. In our case, the modified PR can effectively treat relatively complex retinal detachment cases with multiple breaks spanning more than 1-clock hour. For instance, our reported case had multiple retinal breaks in the right eye, close to a 3-clock-hour position. The use of low-concentration 14% C_3_F_8_ gas can maintain a relatively large gas bubble shortly after injection. By employing a sequential alternating head position maneuver, the multiple retinal breaks can be individually closed. In the case reported by Rubin et al. (7) successful PR surgery was also performed using 0.4 ml of 100% C_3_F_8_ gas; however, new retinal breaks appeared two months postoperatively, possibly due to the prolonged presence of C_3_F8 gas bubble that lead to vitreoretinal traction in the eye.

Traditional PR is typically performed in an office-based setting, with patients holding a fixed head position at home for over 48 h until the next follow-up. However, the efficacy of subretinal fluid absorption and the reliability of maintaining the correct head position during this home period are unknown. In our modified PR procedure for bilateral RRD, hospitalization was deemed necessary. This allowed for timely adjustments to the head position after gas injection and the application of laser photocoagulation upon retinal reattachment. Additionally, ultrawide field fundus photography was employed to document the recovery of retinal detachment and the remaining volume of gas bubbles. This documentation facilitated the proper adjustment of the patient’s head position based on subretinal fluid absorption and enabled the detection of any new retinal breaks during the head position maneuver.

In conclusion, this case report presents a novel modified PR technique for the management of simultaneous bilateral RRD. This innovative approach, incorporating the use of a low concentration of expansile gas and a strategic head position maneuver, demonstrates significant efficacy in a challenging clinical scenario.

## Data availability statement

The raw data supporting the conclusions of this article will be made available by the authors, without undue reservation.

## Ethics statement

The studies involving humans were approved by the Ethics Committee of Taizhou Hospital of Zhejiang Province. The studies were conducted in accordance with the local legislation and institutional requirements. The participants provided their written informed consent to participate in this study. Written informed consent was obtained from the individual(s) for the publication of any potentially identifiable images or data included in this article.

## Author contributions

SG: Conceptualization, Data curation, Investigation, Writing – original draft, Writing – review & editing. TZ: Conceptualization, Data curation, Investigation, Writing – original draft, Writing – review & editing. XZ: Data curation, Writing – review & editing. GL: Writing – review & editing. ZX: Writing – review & editing. EL: Conceptualization, Funding acquisition, Investigation, Project administration, Supervision, Writing – original draft, Writing – review & editing.
